# Association of Interleukin-10 Polymorphism (rs1800896, rs1800871, and rs1800872) With Breast Cancer Risk: An Updated Meta-Analysis Based on Different Ethnic Groups

**DOI:** 10.3389/fgene.2022.829283

**Published:** 2022-02-04

**Authors:** Lijun Li, Wei Xiong, Donghua Li, Jiangang Cao

**Affiliations:** ^1^ The Second Affiliated Hospital, Department of Pharmacy, Hengyang Medical School, University of South China, Hengyang, China; ^2^ The Second Affiliated Hospital, Department of Breast and Thyroid Surgical, Hengyang Medical School, University of South China, Hengyang, China; ^3^ The Affiliated Nanhua Hospital, Clinical Research Institute, Hengyang Medical School, University of South China, Hengyang, China

**Keywords:** interleukin-10, gene polymorphism, breast cancer, meta-analysis, species variation

## Abstract

**Background:** The interleukin10 (IL-10) gene polymorphisms have been indicated to be associated with breast cancer (BC) risk, but the findings are still controversial. To derive a more precise evaluation, we performed a comprehensive meta-analysis.

**Methods:** A systematic literature search was conducted using PubMed, Embase, CNKI, China biomedical (CBM), and Google Scholar to 29 March 2020. Revman5.3 and Stata 12.0 software analyzed the data, and the strength of the association was identified using the odds ratio (OR) and the corresponding 95% confidence interval (CI).

**Results:** A total of 23 studies (7,250 cancer cases and 7,675 case-free controls) were included in this meta-analysis. The results show that IL-10 gene polymorphisms were significantly correlated with BC risk based on subgroup analysis by ethnicity. The IL-10 rs1800896 polymorphism was significantly associated with the risk of BC in Asians (G vs. A: OR = 0.78, 95% CI = 0.65–0.95, *p* = 0.01; GG vs. AA: OR = 0.51, 95% CI = 0.31–0.84, *p* = 0.007; GA vs. AA: OR = 0.6, 95% CI = 0.44–0.81, *p* = 0.0009; GG + GA vs. AA: OR = 0.6, 95% CI = 0.45–0.81, *p* = 0.0007); Moreover, an increased BC risk in Asians were also associated with the IL-10 rs1800872 polymorphism (AA vs CC: OR = 0.74, 95% CI = 0.55–0.99, *p* = 0.04; A vs C: OR = 0.85, 95% CI = 0.74–0.98, *p* = 0.03). In addition, The IL-10 rs1800871 (CT vs. TT: OR = 1.8, 95% CI = 1.03–3.13, *p* = 0.04) and rs1800872 polymorphism (A vs C: OR = 0.65, 95% CI 0.43–0.98, *p* = 0.04) were associated with BC risk in Caucasians.

**Conclusion:** Collectively, this meta-analysis demonstrated that IL-10 rs1800896 and rs1800872 (AA vs. CC; A vs. C) polymorphisms significantly increased the risk of BC in Asians, while the rs1800871 and rs1800872 (A vs. C) were associated with the risk of BC in Caucasians. Therefore, this may provide new ideas for predicting and diagnosing BC susceptibility through the detection of IL-10 gene polymorphism.

**Systematic Review Registration:** [https://www.crd.york.ac.uk/ PROSPERO], identifier [CRD42021266635].

## Introduction

Breast cancer (BC) is the leading cause of female cancer-related death worldwide and is one of the most common cancer forms (Anastasiadi et al., 2017). BC incidence varies widely, ranging from 27/100,000^2^ (Central-East Asia and Africa) to 85–94/100,000^2^ (Australia, North America, and Western Europe). And the incidence of BC in France is the highest in Europe (Sancho-Garnier and Colonna, 2019). In Asian countries, the incidence rate of BC has also been increasing rapidly (Mubarik et al., 2020; Oblak et al., 2020). The pathogenesis of BC is multifactorial. Hereditary BC accounts for only 5–10% of all BC cases and germline mutations, with the two significant BC susceptibility genes, *BRCA1* and *BRCA2* is responsible for approximately 2–3% of all cases (Kwong et al., 2016). Besides gene tests for identifying high-risk BRCA1 or BRCA2 mutations carriers (Ha et al., 2017), the ability to predict BC development is not well established yet. Although genetic, environmental, and lifestyle factors are associated with BC occurrence, the biological mechanism that causes BC remains unclear.

Inflammation plays a significant role in BC development and is an important part of the BC microenvironment (Mohamed et al., 2018). Interleukin-10 (IL-10) is an important anti-inflammatory and immunomodulatory cytokine in the human immune response. IL-10 is located on chromosome 1 (1q31-1q32), composed of five exons and four introns (Roh et al., 2002). Single nucleotide polymorphism (SNP) is the most common genetic variation. In the SNP database (http://www.ncbi.nlm.nih.gov/snp), three promoter SNPs of IL-10, rs1800896 (-1082A/G), rs1800871 (-819T/C), and rs1800872 (-592A/C) were extensively investigated in many diseases. Because they might affect IL-10 gene transcription and translation, resulting in abnormal cell proliferation and cancer development (Howell and Rose-Zerilli, 2007). The possible mechanism is that IL-10 is activated by the Janus kinase (JAK)/signal transducer and activator of transcription (STAT) signaling pathways through its receptor IL-10 R1 which binds to STAT3. Then STAT3 is translocated into the nucleus, where it binds to STAT-binding elements in the promoters of proliferation-related genes. It has been reported that IL-10 gene polymorphism plays an important role in the occurrence and development of cancers such as BC, gastric cancer, lung cancer (Bhattacharjee et al., 2016; Chen et al., 2019; Zhao et al., 2019). And some studies reported the high IL-10 expression levels in the BC paraffin section and its expression is correlated with worse outcomes in patients with malignant tumors (Li et al., 2014; Zhao et al., 2015).

In recent years, several studies have reported the relationship between IL-10 polymorphisms and BC susceptibility. A study found that: rs1800896 (-1082A/G) polymorphism was correlated with cancer staging and associated with the progression of BC at AA genotype (Abedinzadeh et al., 2018). In the research based on Caucasians, it was found that there was a significant association between the IL10-1082 G/G genotype and the increased risk of BC (Zhu et al., 2020). Another study found that the rs1800871 (-819T/C) polymorphism increased the risk of BC in Han Chinese women (Li et al., 2020). And a study shows the rare allele of rs1800872 (-592A/C) polymorphism may be a potential prognostic indicator of disease-free survival in BC patients (Gerger et al., 2010). These suggest that IL-10 gene polymorphism may affect the risk of human BC (Setrerrahmane and Xu, 2017). However, these results are inconsistent. Moreover, IL-10 polymorphism and BC susceptibility studies are constantly updated, and adjustments vary between included studies based on race, age, lifestyle, and other covariates (Patricia Gallegos-Arreola et al., 2019). Considering the critical role of IL-10 in the development of BC, we conducted this systematic review. And compared with previous meta-analyses, we comprehensively included the latest relevant studies to evaluate the association of IL-10 rs1800896, rs1800871, and rs1800872 polymorphisms with the risk of BC in different ethnic groups. It will provide theoretical evidence for the genetic mechanism of BC.

## Methods

This meta-analysis was conducted according to the PRISMA reporting criteria (Moher et al., 2009).

### Search Strategy

Research articles on the relationship between IL-10 gene polymorphisms and BC risk were searched in different databases, including PubMed, Web of Knowledge, Embase, CNKI, CBM, and Google Scholar up to 29 March 2020. And we retrieved with the keywords: (“breast cancer” or “breast tumor” or “breast neoplasm” or “malignant breast tumor” or “breast carcinoma”) and (“Interleukin-10” or “IL-10”) and (“polymorphism” or “SNP” or “single nucleotide polymorphism” or “variation” or “mutation”).

### Inclusion and Exclusion Criteria

Inclusion criteria: (1) Clinical BC patients were selected as the case group and healthy people as the control group; (2) Case-control or cohort studies about associations between IL-10 gene polymorphism and BC in humans; (3) Full manuscript in English or Chinese is retrievable; (4) Reporting the number of cases and controls for each genotype and detailed genotyping data, or knowing the odds ratio (OR) helped to calculate the 95% confidence interval (CI).

Exclusion criteria: (1) Abstracts, reviews; (2) Studies on the relationship between IL-10 gene polymorphism and prognosis of BC; (3) studies on the apparent imbalance of baseline between the case group and control group; (4) The cases and control sources were not provided; (5) Repeatedly published literature. If multiple studies from the same case series were available, the one including the most individuals were used in the analysis.

### Data Extraction

Two researchers selected the literature according to the inclusion and exclusion criteria, extracted the data, and cross-checked them independently into a standard data collection form. If there were any disputes, we would reach an agreement by discussion or by a third party and strive to reach a consensus on each project. Data were collected from each article included: the first author, year of publication, study location, type of study, ethnicity (classified as Asian, Caucasian, or mixed descent), total number of cases and controls, genotype frequency, genotype detection method, and the source of authority.

### Sensitivity Analysis

Sensitivity analysis was performed to assess the stability of the results. The Funnel plot, Begg’s test, and Egger’s test were used to evaluate publication bias. RevMan5.3 and Stata 12.0 software was used for the above statistical analysis.

### Statistical Analysis

The correlation between IL-10 gene polymorphisms and BC risk was evaluated by OR and 95% CI as the effect size. 95% CI without one and *P*(OR) < 0.05 was considered statistically significant. The Z-test determines the significance of the OR value. The effects of heterogeneity were quantified by I^2^ and *P*(H) values. In addition, the I^2^ value is used to quantify the degree of heterogeneity (I^2^ < 25%: low/no heterogeneity; 25 < I^2^ < 75%: moderate heterogeneity; I^2^ > 75%: extreme high heterogeneity). The fixed-effect model is adopted when the I^2^ < 25%; otherwise, the random effect model is adopted. We further carried out subgroup analyses by ethnicity to get ethnic-specific results.

## Results

### Search Results

**FIGURE 1:**
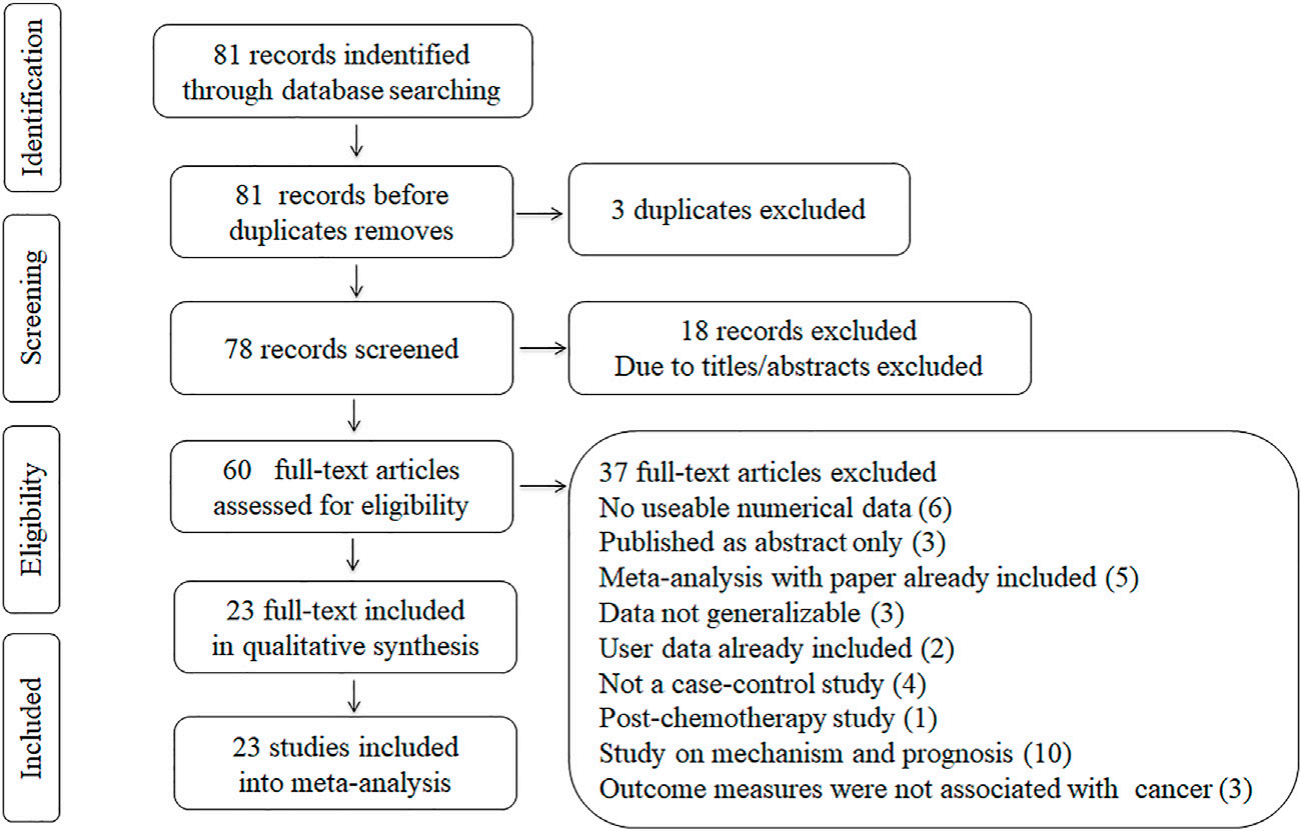
Flowchart of study selection for the present study.

We had a total of 78 articles after removing three duplicated pieces. After the layer-by-layer screening, a total of 23 articles finally met the criteria for inclusion in this meta-analysis. Eligible papers were published between 2004 and 2019. This meta-analysis updated three 2019 case-control studies compared to previous meta-analyses (Dai et al., 2014; Abedinzadeh et al., 2018; Moghimi et al., 2018). A flow diagram schematizing the inclusion and exclusion process of identified articles with the inclusion criteria is presented in Figure 1.

### Data Extraction and Quality Assessment

The 23 eligible articles had a total sample size of 14,925 participants, including 7,250 BC patients and 7,675 healthy controls (Smith et al., 2004; Abdolrahim-Zadeh et al., 2005; Guzowski et al., 2005; Langsenlehner et al., 2005; Balasubramanian et al., 2006; Onay et al., 2006; Scola et al., 2006; Gonullu et al., 2007; Pharoah et al., 2007; Kong et al., 2010; He et al., 2012; Pooja et al., 2012; Meijiang, 2014; Wang et al., 2014; Vinod et al., 2015; AlSuhaibani et al., 2016; Atoum, 2016; Maruthi et al., 2017; Sabet et al., 2017; Tian et al., 2017; Azher et al., 2019; Fanyu et al., 2019; Patricia Gallegos-Arreola et al., 2019). The samples were involved in three IL-10 polymorphism sites: rs1800896, rs1800871, and rs1800872. There were 17 studies on rs1800896 (3,308 cases and 3,425 controls), twelve studies on rs1800871 (2,530 cases and 2,698 controls), and 13 studies on rs1800872 (4,702 cases and 4,818 controls). Ten studies were based on Caucasians, six were based on Asians, and the remaining seven studies were mixed-race in the 23 criteria studies. Of these studies, eighteen were hospital-based, and five were population-based. The Newcastle-Ottawa Scale (NOS) was used to assess the quality of the included articles (Stang, 2010). And NOS scores ranged from zero to nine. We considered the study’s methodological quality good if the score was ≥ seven. Two authors independently completed our data extraction and quality evaluation. Table 1 and Table 2 show the basic characteristics of the included literature, the distribution of polymorphisms at the studied gene sites, allele frequency, and the quality assessment of the included studies.

**TABLE 1:** Characteristics of the studies included in the meta-analysis.

First author	Year	Country	Ethnicity	Genotyping method		SOC	Case/control	Study design	SNP No.	NOS score
Gallegos-Arreola	2019	Mexican	Mixed	PCR-RFLP	HB		368/320	CC	3	8
Al-Ankoshy	2019	Iraq	Caucasian	PCR–SSP	HB		70/70	CC	1	8
Zeng	2019	China	Asian	PCR-RFLP	HB		208/215	CC	3	8
Sabet	2017	Egypt	Caucasian	PCR-RFLP	HB		105/50	CC	1,2,3	7
Tian	2017	China	Asian	Mass ARRAY	PB		312/312	CC	1,2,3	7
Maruthi	2017	India	Mixed	PCR-RFLP	HB		285/285	CC	1	7
Atoum	2016	Jordan	Mixed	PCR-RFLP	HB		202/210	CC	1,2,3	7
Alsuhaibani	2016	Egypt	Caucasian	PCR-RFLP	HB		80/80	CC	1	7
Vinod	2015	India	Mixed	ASPCR	HB		125/160	CC	1	8
Li	2014	China	Asian	PCR–SSP	PB		128/128	CC	1,2	7
Wang	2014	China	Asian	PCR-RFLP	HB		474/501	CC	2	8
Pooja	2012	India	Mixed	PCR-RFLP	PB		200/200	CC	1,2,3	7
He	2012	China	Asian	MALDI-TOF MS	HB		347/500	CC	2	7
Kong2010	2010	China	Asian	PCR-RFLP	HB		315/322	CC	1,2,3	7
Pharoah	2007	European	Caucasian	TaqMan	PB		2045/2218	CC	3	8
Gonullu	2007	Turkey	Caucasian	Mass ARRAY	HB		38/24	CC	1,2,3	7
Scola	2006	Italy	Caucasian	PCR-RFLP	HB		84/106	CC	1,2,3	7
Onay	2006	Canada	Mixed	TaqMan	PB		398/372	CC	1	8
Balasu bramanian	2006	United Kingdom	Caucasian	TaqMan	HB		497/498	CC	1	7
Guzowski	2005	America	Mixed	DHPLC	HB		50/25	CC	1,2,3	7
Langsenlehner	2005	Australia	Caucasian	TaqMan	PB		500/496	CC	3	8
Abdolrahim	2005	Iran	Caucasian	PCR-RFLP	HB		275/320	CC	1,2,3	8
Smith	2004	United Kingdom	Caucasian	ARMS-PCR	HB		144/263	CC	1	8

SOC, source of controls; HB: hospital-based; PB, population-based; CC, case–control; PCR, polymerase chain reaction; RFLP, restriction fragment length polymorphism; DHPLC, denaturing highperformance liquid chromatography; EPIC, European Prospective Investigation of Cancer (a prospective study of diet and cancer being carried out in nine European countries); ASPCR, allele-specific PCR; SNP, single-nucleotide polymorphisms; SNP No. 1, - 1082A > G (rs1800896); 2: - 819T > C (rs1800871); 3, - 592A > C (rs1800872); NOS, Newcastle-ottawa scale.

**TABLE 2:** IL-10 polymorphisms genotype distribution and allele frequency in cases and controls.

First author	Case	Control	Cases					Control					MAF
			Genotypes			Alleles		Genotypes			Alleles		
-1082A > G rs1800896			AA	AC	CC	A	C	AA	AC	CC	A	C	
Atoum (2016)	202	210	157	29	16	343	61	151	42	17	344	76	0.181
AlSuhaibani et al. (2016)	80	80	16	47	17	79	81	14	50	16	78	82	0.512
Vinod et al. (2015)	125	160	76	31	18	183	67	67	78	15	212	108	0.337
Pooja et al. (2012)	200	200	132	60	8	324	76	145	50	5	34	60	0.638
Kong et al. (2010)	315	322	285	29	1	599	31	285	35	2	605	39	0.061
Gonullu et al. (2007)	38	24	13	22	3	48	28	16	7	1	39	9	0.187
Guzowski et al. (2005)	50	25	10	28	12	48	52	9	12	4	30	20	0.400
Sabet et al. (2017)	105	50	15	41	49	71	139	27	21	2	75	25	0.250
Tian et al. (2017)	312	312	51	132	129	234	390	27	154	131	208	416	0.666
Abdolrahim-Zadeh et al. (2005)	275	320	119	116	40	171	373	146	125	49	417	223	0.348
Scola et al. (2006)	84	106	28	40	16	96	72	40	45	21	125	87	0.410
Onay et al. (2006)	398	372	90	205	103	385	411	107	194	71	408	336	0.451
Balasubramanian et al. (2006)	497	498	121	253	123	499	495	117	260	121	494	502	0.504
Maruthi et al. (2017)	285	285	80	146	59	262	308	89	159	37	234	336	0.589
Azher et al. (2019)	70	70	36	10	24	97	43	16	17	37	44	96	0.690
Smith et al. (2004)	144	263	32	58	39	136	122	46	120	57	250	276	0.524
Li et al. (2014)	128	128	96	30	2	222	34	80	44	4	204	52	0.203
-819T > C (rs1800871)			TT	TC	CC	T	C	TT	TC	CC	T	C	
Atoum (2016)	202	210	88	47	67	223	181	93	41	76	227	193	0.459
Wang et al. (2014)	474	501	90	198	186	378	570	48	219	234	315	687	0.685
Pooja et al. (2012)	200	200	54	92	54	200	200	65	78	57	208	192	0.480
Kong et al. (2010)	315	322	119	135	61	273	257	134	131	57	399	245	0.380
Gonullu et al. (2007)	38	24	5	17	16	27	49	4	10	10	18	30	0.625
Guzowski et al. (2005)	50	25	3	19	28	25	75	1	10	14	12	38	0.760
Sabet et al. (2017)	105	50	16	47	42	79	131	26	22	2	74	26	0.260
Tian et al. (2017)	312	312	124	141	47	389	235	144	128	40	416	208	0.333
Abdolrahim-Zadeh et al. (2005)	275	320	129	120	26	375	172	166	122	32	454	186	0.290
He et al. (2012)	347	500	177	141	29	495	199	229	223	44	681	311	0.313
Scola et al. (2006)	84	106	5	30	49	40	128	12	35	59	59	177	0.721
Li et al. (2014)	128	128	105	22	1	232	23	96	28	4	220	36	0.203
-592C > A (rs1800872)			AA	AC	CC	A	C	AA	AC	CC	A	C	
Patricia Gallegos-Arreola et al. (2019)	368	320	42	154	172	238	498	11	100	209	122	518	0.190
Zeng et al. (2019)	208	215	10	88	110	108	308	22	95	98	139	291	0.323
Atoum (2016)	202	210	76	84	42	236	168	79	91	40	249	171	0.593
Pooja et al. (2012)	200	200	45	67	88	157	243	38	84	78	160	240	0.400
Kong et al. (2010)	315	322	119	135	61	373	257	134	131	57	399	245	0.620
Gonullu et al. (2007)	38	24	5	17	16	27	49	4	10	10	18	30	0.375
Guzowski et al. (2005)	50	25	3	17	30	23	77	3	10	12	16	34	0.320
Langsenlehner et al. (2005)	500	496	21	210	269	252	748	36	199	261	271	721	0.273
Sabet et al. (2017)	105	50	4	36	65	42	166	31	16	6	78	28	0.736
Tian et al. (2017)	312	312	131	130	51	392	232	141	127	44	409	215	0.655
Abdolrahim-Zadeh et al. (2005)	275	320	27	100	148	154	396	29	132	159	190	450	0.297
Scola et al. (2006)	84	106	5	30	49	40	128	12	35	59	59	153	0.278
Pharoah et al. (2007)	2045	218	116	679	1,251	367	3,181	116	764	1,338	996	3,440	0.225

MAFs: minor allele frequencies.

### Meta-Analysis Results

**FIGURE 2:**
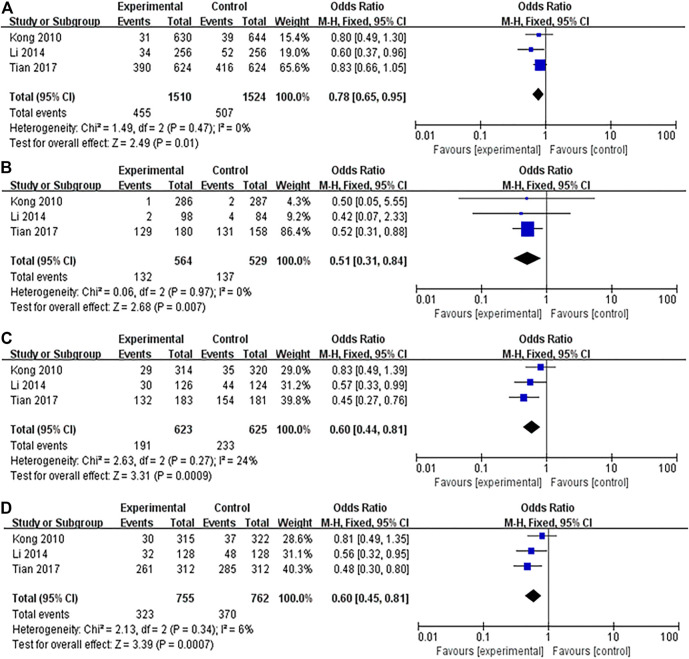
Forest plots showed a significant association of IL-10 rs1800896 polymorphism and breast cancer risk in Asians. **(A)** (allele model: G vs. A); **(B)** (homozygous model: GG vs. AA); **(C)** (heterozygous model: GA vs. AA); **(D)** (dominant model: GG + GA vs. AA). The squares and horizontal lines correspond to the study-specific odds ratio (OR) and 95% confidence interval (CI). The area of the squares reflects the weight (inverse of the variance). The diamond represents the summary OR and 95% CI.

**FIGURE 3:**
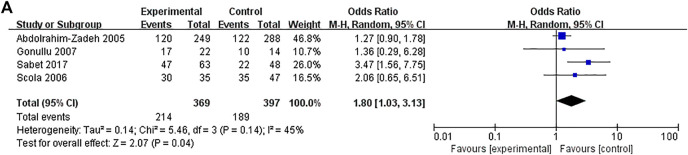
Forest plots showed a significant association of IL-10 rs1800871 polymorphism and breast cancer risk in the Caucasians (heterozygous model: CT vs. TT). The squares and horizontal lines correspond to the study-specific odds ratio (OR) and 95% confidence interval (CI). The area of the squares reflects the weight (inverse of the variance). The diamond represents the summary OR and 95% CI.

**FIGURE 4:**
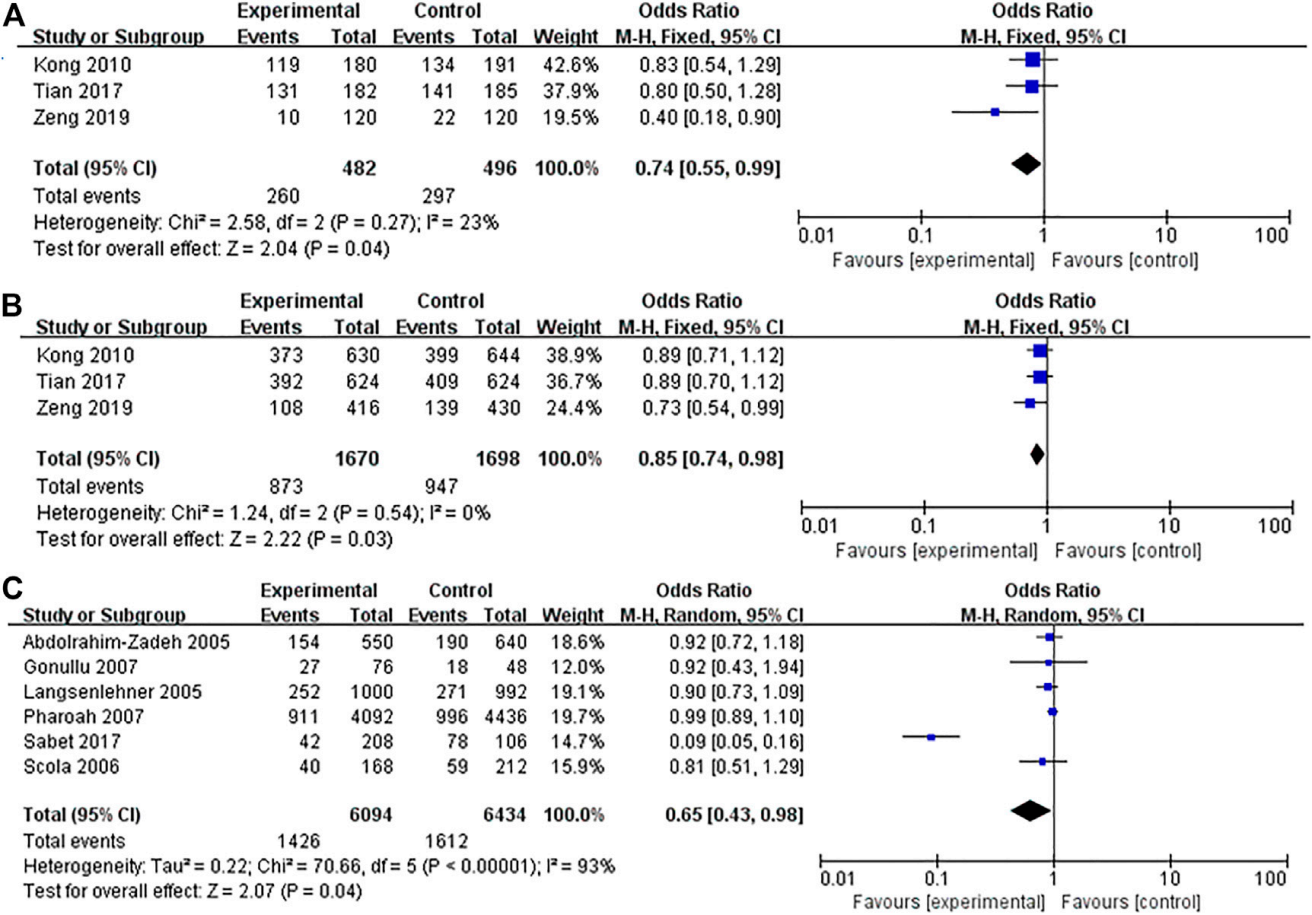
Forest plots showed a significant association of IL-10 rs1800872 polymorphism and breast cancer risk in Asians and Caucasians. **(A)** IL-10 rs1800872 polymorphism in Asians (homozygous model: AA vs. CC); **(B)** IL-10 rs1800872 polymorphism in Asians (allele model: A vs. C); **(C)** IL-10 rs1800872 polymorphism in Caucasians (allele model: A vs. C). The squares and horizontal lines correspond to the study-specific odds ratio (OR) and 95% confidence interval (CI). The area of the squares reflects the weight (inverse of the variance). The diamond represents the summary OR and 95% CI.

**FIGURE 5:**
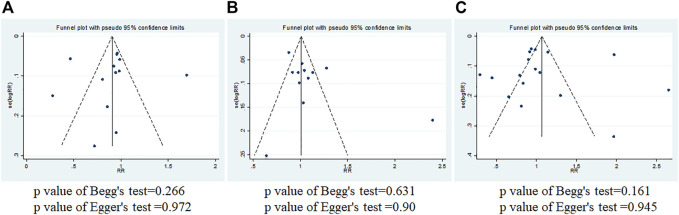
Begg’s and Egger’s funnel plots of IL-10 gene polymorphism and breast cancer risk for publication bias test. **(A)** rs1800896; **(B)** rs1800871; **(C)** rs1800872.

The association between IL-10 gene polymorphisms (rs1800896, rs1800871, and rs1800872) and BC is shown in Table 3 and Figures 2–5. Squares and horizontal lines correspond to study-specific OR and 95% CI. The area of a square reflects the weight (inversely proportional to the variance). The diamond represents the sum of OR and 95% CI.

**TABLE 3:** Results of the association of IL-10 polymorphisms with BC risk.

Subgroup	Genetic model	Type of model	Heterogeneity		Odds Ratio			
			I^2^ (%)	PH	OR	95% CI	Z test	POR
*rs1800896*								
Overall	G vs. A	Random	82	<0.00001	1.06	0.87–1.28	0.56	0.57
	GG vs. AA	Random	71	<0.00001	1.14	0.81–1.59	0.73	0.46
	GA vs. AA	Random	73	<0.00001	0.91	0.71–1.17	0.74	0.46
	GG + GA vs. AA	Random	79	<0.00001	0.99	0.76–1.28	0.11	0.92
	GG vs. GA + AA	Random	53	0.006	1.18	0.94–1.48	1.45	0.15
Ethnicity								
Asian	G vs. A	Fixed	0	0.47	0.78	0.65–0.95	2.49	**0.01**
	GG vs. AA	Fixed	0	0.97	0.51	0.31–0.84	2.68	**0.007**
	GA vs. AA	Fixed	24	0.27	0.6	0.44–0.81	3.31	**0.0009**
	GG + GA vs. AA	Fixed	6	0.34	0.6	0.45–0.81	3.39	**0.0007**
	GG vs. GA + AA	Fixed	0	0.66	0.94	0.69–1.28	0.39	0.7
Caucasian	G vs. A	Random	0.0089	<0.00001	1.19	0.83–1.72	0.94	0.35
	GG vs. AA	Random	0.008	0.00001	1.21	0.67–2.16	0.63	0.53
	GA vs. AA	Random	72	0.0008	1.09	0.73–1.62	0.43	0.67
	GG + GA vs. AA	Random	84	<0.00001	1.19	0.74–1.91	0.7	0.48
	GG vs. GA + AA	Random	70	0.002	1.14	0.75–1.72	0.61	0.55
*rs1800871*								
Overall	C vs. T	Random	84	<0.00001	1.11	088–1.39	0.87	0.39
	CC vs. TT	Random	75	<0.00001	1.12	0.75–1.66	0.55	0.58
	CT vs. TT	Random	67	0.0004	1.11	0.85–1.44	0.77	0.44
	CC + CT vs. TT	Random	78	<0.00001	1.12	0.84–1.50	0.77	0.44
	CC vs. CT + TT	Random	50	0.03	1	0.80–1.25	0.02	0.98
Ethnicity								
Asian	C vs. T	Random	89	<0.00001	0.94	0.68–1.31	0.35	0.72
	CC vs. TT	Random	79	0.0008	0.81	0.48–1.40	0.74	0.46
	CT vs. TT	Random	76	0.002	0.86	0.61–1.21	0.88	0.38
	CC + CT vs. TT	Random	82	0.0001	0.84	0.58–1.22	0.93	0.35
	CC vs. CT + TT	Random	38	0.17	0.93	0.72–1.20	0.59	0.56
Caucasian	C vs. T	Random	88	<0.0001	1.57	0.81–3.05	1.33	0.18
	CC vs. TT	Random	84	0.0004	1.11	0.85–1.44	0.77	0.44
	CT vs. TT	Random	45	0.14	1.8	1.03–3.13	2.07	**0.04**
	CC + CT vs. TT	Random	79	0.002	2.13	0.89–5.13	1.69	0.09
	CC vs. CT + TT	Random	78	0.003	1.64	0.69–3.90	1.12	0.26
*rs1800872*								
Overall	A vs. C	Random	89	<0.00001	0.82	0.66–1.03	1.7	0.09
	AA vs. CC	Random	83	<0.00001	0.71	0.46–1.08	1.6	0.11
	AC vs. CC	Random	59	0.004	0.93	0.78–1.11	0.8	0.43
	AA + AC vs. CC	Random	79	0.00001	0.86	0.68–1.08	1.31	0.19
	AA vs. AC + CC	Random	60	0.004	0.95	0.75–1.20	0.44	0.66
Ethnicity								
Asian	A vs. C	Fixed	0	0.54	0.85	0.74–0.98	2.22	**0.03**
	AA vs. CC	Fixed	23	0.27	0.74	0.55–0.99	2.04	**0.04**
	AC vs. CC	Fixed	0	0.88	0.88	0.69–1.13	0.96	0.34
	AA + AC vs. CC	Fixed	0	0.81	0.82	0.65–1.04	1.65	0.1
	AA vs. AC + CC	Fixed	25	0.26	0.82	0.66–1.01	1.84	0.07
Caucasian	A vs. C	Random	93	<0.00001	0.65	0.43–0.98	2.07	**0.04**
	AA vs. CC	Random	89	<0.00001	0.43	0.19–1.00	1.96	0.05
	AC vs. CC	Random	48	0.09	0.89	0.72–1.11	1.02	0.31
	AA + AC vs. CC	Random	82	<0.00001	0.74	0.52–1.05	1.69	0.09
	AA vs. AC + CC	Random	36	0.18	0.87	0.62–1.21	0.84	0.4

OR, odds ratio; PH, *p* value of Heterogeneity; CI, confidence intervals; POR, *p* value of odds ratio. *p* value, significant at <0.05. Bold numbers denote statistical significance.

### Correlation Between rs1800896 Polymorphism and Breast Cancer

A total of 17 studies were conducted on the association between IL-10 rs1800896 polymorphism and BC risk, with a total sample size of 6,733 cases, including 3,308 patients and 3,425 healthy controls. Overall population heterogeneity test I^2^ was 82%. The random-effect model results showed that the comparison results of the five gene models showed no statistical significance between rs1800896 polymorphism and BC (Table 3). Subgroups by ethnicity showed that under the four genetic models (allele G vs. A: OR = 0.78, 95% CI = 0.65–0.95, *p* = 0.01; homozygous GG vs. AA: OR = 0.51, 95% CI = 0.31–0.84, *p* = 0.007; heterozygous GA vs. AA: OR = 0.6, 95% CI = 0.44–0.81, *p* = 0.0009; dominant GG + GA vs. AA: OR = 0.6, 95% CI = 0.45–0.81, *p* = 0.0007) (Figures 2A–D), rs1800896 polymorphism was significantly associated with BC risk in Asians. This result suggests that ethnicity is likely to be the source of heterogeneity, and the rs1800896 polymorphism is significantly associated with the BC risk in Asians.

### Correlation Between rs1800871 Polymorphism and Breast Cancer

A total of 12 studies with 2,530 patients and 2,698 controls evaluated the strength of the association between the IL-10 rs1800871 polymorphism and BC. There was no association between BC risk and rs1800871 polymorphism in any genetic model in the overall population. However, when stratified by ethnicity, the rs1800871 polymorphism was associated with BC risk in the heterozygous model in Caucasians (CT vs. TT: OR = 1.8, 95% CI = 1.03–3.13, *p* = 0.04) (Figure 3). This result indicates that Caucasians with the rs1800871 heterozygous model are more likely to develop BC than individuals with other genotypes.

### Correlation Between rs1800872 Polymorphism and Breast Cancer

Thirteen studies (4,702 cases and 4,818 controls) assessed the strength of the association between IL-10 rs1800872 polymorphism and BC susceptibility. As shown in Table 3, the five gene model comparison results showed that the association between IL-10 rs1800872 polymorphism and BC in the overall population was not statistically significant. However, after stratification by ethnicity, the homozygous model of rs1800872 polymorphism was associated with BC risk in Asians (AA vs. CC: OR = 0.74, 95% CI = 0.55–0.99, *p* = 0.04) (Figure 4A). Allele model of rs1800872 polymorphism was associated with the risk of BC in Asians (A vs. C: OR = 0.85, 95% CI = 0.74–0.98, *p* = 0.03) (Figure 4B) and Caucasians (A vs. C: OR = 0.65, 95% CI = 0.43–0.98, *p* = 0.04) (Figure 4C).

### Publication Bias

Funnel plot, Begg’s test, and Egger’s test were used to evaluate the publication bias (Stata12.0). As shown in Figure 5, the funnel plot was essentially symmetrical, and the *p* values of Begg’s test and Egger’s test are all greater than 0.05. It was indicated that there was almost no obvious publication bias at the three loci.

## Discussion

IL-10, known initially as cytokine synthesis inhibitory factor (CSIF), is a potent anti-inflammatory cytokine. IL-10 can stimulate the expression of carboxypeptidase B2 (CPB2) in inflammatory BC cells. Thus it increases the cancer cells’ aggressiveness (Mohamed et al., 2018). Moreover, IL-10 is involved in the abnormal proliferation of breast ducts and lobules and stimulates mitotic activity, leading to increased cancer risk (Kong et al., 2010; Moghimi et al., 2018). IL-10 can also induce tumor progression by inhibiting many cytokines such as IL-1a, IL-1b, IL-6, IL-8, IL-12, and IL-18. And IL-10 gene silencing down-regulates the expression of phosphoinositide 3-kinase (PI3K)/protein kinase B (AKT) and B cell lymphoma 2 (Bcl2) and increases the expression levels of BCL2 binding component 3(BBC3), Bax, and caspase3 (Alotaibi et al., 2018). Studies on the mechanism of IL-10 promoting BC have shown that the production of IL-10 may represent a new escape mechanism for BC cells to escape the destruction of the immune system. It might be closely related to the fact that polymorphic variations in the promoter sequences of the IL-10 gene might influence the gene expression and consequently play a specific role in susceptibility and the clinical course of BC. The IL-10 promoter region polymorphisms affected IL-10 gene transcription and translation, resulting in abnormal cell proliferation and cancer development (Moghimi et al., 2018; Sheikhpour et al., 2018).

Studies have shown that the three most common single nucleotide polymorphisms (SNPs) play an important role in regulating IL-10 activity. They are located at the transcriptional starting point of rs1800896 (-1082A/G), rs1800871 (-819T/C), and rs1800872 (-592A/C). And they encode high (GCC), medium (ACC), and low (ATA) expression of IL-10, respectively (Westendorp et al., 1997; Zupin et al., 2014; Hofmann et al., 2018). Several other polymorphic loci of IL-10 (rs1800890, rs6703630, and rs6693899) are also controversial, but few relevant studies are present. Many studies have reported the relationship between race and IL-10 gene polymorphism and BC risk in recent years. For example, the IL-10 rs1800872 polymorphism was associated with BC susceptibility in the Mexican population (Patricia Gallegos-Arreola et al., 2019). Also, the mutant allele and genotypes of IL-10 rs1800896 were significantly associated with Indian postmenopausal BC (Pooja et al., 2012). Since the IL-10 gene polymorphisms were associated with the risk of BC, we hypothesized that race is the key to the association between IL-10 gene polymorphisms and BC. This meta-analysis conducted the most comprehensive analysis of the relationship between three IL-10 polymorphisms (rs1800896, rs1800871, and rs1800872) and the BC risk of different races. In a subgroup analysis by ethnicity (Asian and Caucasian/mixed race), the three IL-10 polymorphisms (rs1800896, rs1800871, and rs1800872) were significantly associated with BC. It showed that rs1800896 (allele G vs. A: OR = 0.78, 95% CI = 0.65–0.95, *p* = 0.01; homozygous GG vs. AA: OR = 0.51, 95% CI = 0.31–0.84, *p* = 0.007; heterozygous GA vs. AA: OR = 0.6, 95% CI = 0.44–0.81, *p* = 0.0009; dominant GG + GA vs. AA: OR = 0.6, 95% CI = 0.45–0.81, *p* = 0.0007) were significantly correlated with BC risk in Asians. The rs1800871 heterozygote model (CT vs. TT: OR = 1.8, 95% CI = 1.03–3.13, *p* = 0.04) was associated with BC risk in Caucasians. The rs1800872 homozygous model (AA vs CC: OR = 0.74, 95% CI = 0.55–0.99, *p* = 0.04) was associated with BC risk in Asians, and the allelic model (A vs. C: OR = 0.85, 95% CI = 0.74–0.98, *p* = 0.03) was associated with BC risk in Asians and Caucasians (A vs C: OR = 0.65, 95% = CI 0.43–0.98, *p* = 0.04). The above results indicate that the ethnic subgroup of IL-10 gene polymorphisms is the key factor affecting the susceptibility to BC. It is consistent with the results of previous studies: the relationship between IL-10 gene polymorphism and BC risk is strongly associated with ethnicity (Patricia Gallegos-Arreola et al., 2019).

Previously, three researchers (Dai et al., 2014; Abedinzadeh et al., 2018; Moghimi et al., 2018) have analyzed the correlation between IL-10 gene polymorphisms and BC risk, but their analysis is not comprehensive enough. Because there are few studies included and the ethnic division is not accurate enough in their articles. In addition, *Xu* and *Wang* did a meta-analysis on the relationship between various interleukins and BC. Still, their correlations between IL-10 gene polymorphisms and BC risk were inconsistent with ours (Xu and Wang, 2020). It may be related to the different criteria for inclusion and exclusion and quality assessment of the article. Because the quality, quantity, and new studies included in the meta-analysis will directly affect the credibility and stability of the results, we used a broad search strategy to capture all relevant information. This meta-analysis conducted a more comprehensive analysis of the relationship between three IL-10 polymorphisms (rs1800896, rs1800871, and rs1800872) and BC risk by including 23 studies (published between 2004 and 2019) and ruling out the researches with low quality. Moreover, this meta-analysis showed no significant publication bias, and the heterogeneity of the subgroups was small. Sensitivity analysis results were also stable. Therefore, the conclusion of the association between the three IL-10 gene polymorphisms (rs1800896, rs1800871, and rs1800872) and BC in this meta-analysis was reliable and had certain clinical guidance values.

However, this meta-analysis has several limitations that should be acknowledged. Firstly, due to the limited research on the interaction between these three polymorphic sites and their interaction with the environment, it is impossible to estimate the impact of gene-gene and gene-environment interaction on the study results. Secondly, we found that heterogeneity existed in the meta-analysis as indicated by the I^2^ values. Despite using a random-effects model in some studies, the heterogeneity remained. It is predictable because other factors that affect BC should be considered, such as staging and grading of tumors, age, genetic background, environment, and lifestyle. However, due to the lack of some qualified original data, we cannot calculate the impact of these factors on BC. Moreover, in the future we need to consider more factors influencing BC, such as age, menopausal state, environment, and lifestyle factors, to further validate gene-gene and gene-environment interactions on IL-10 polymorphisms and BC risk.

## Conclusion

In summary, this meta-analysis provides a new idea for clinical, genetic, and epidemiological studies of BC. Our results show that alleles, homozygotes, and dominant genotypes of IL-10 rs1800896 are significantly associated with the risk of BC in Asians. The homozygous and allele patterns of rs1800872 increase the risk of BC in Asians, while the heterozygous pattern of rs1800871 and the allele pattern of rs1800872 increase the risk of BC in Caucasians. IL-10 gene polymorphisms may be a key regulator of BC susceptibility. Different ethnic groups can predict BC susceptibility by detecting other IL-10 polymorphisms locus. However, the etiology of BC is complex, so we strongly recommend further genetic association studies to explore the effects of gene-gene interactions on disease susceptibility. Large-scale multicenter studies can be conducted in the future to verify further the results of gene-gene and gene-environment interactions on IL-10 gene polymorphisms and BC risk in different environments.
